# Double ampulla anatomy identified during endoscopic retrograde cholangiopancreatography

**DOI:** 10.1093/jcag/gwaf025

**Published:** 2025-09-20

**Authors:** Scott MacKay, Rachel Sullivan, Jan-Erick Nilsson, Gurpal Sandha

**Affiliations:** Division of Gastroenterology, University of Alberta Hospital, Edmonton, AB T6G 2X8, Canada; Division of Gastroenterology, University of Alberta Hospital, Edmonton, AB T6G 2X8, Canada; Division of Gastroenterology, University of Alberta Hospital, Edmonton, AB T6G 2X8, Canada; Division of Gastroenterology, University of Alberta Hospital, Edmonton, AB T6G 2X8, Canada

## Case presentation

A 48-year-old female with known cholelithiasis presented to hospital with epigastric pain, acute liver enzyme elevation, and hyperbilirubinemia. Endoscopic retrograde cholangiopancreatography (ERCP) was completed and, as the duodenoscope was positioned across the ampulla, 2 ampullary mounds were observed (Figure 1). The upper ampullary mound was successfully cannulated, and sphincterotomy was performed with stone clearance achieved using a balloon extraction catheter. Subsequent MRCP demonstrated variant pancreas divisum anatomy with the dominant continuation of the main pancreatic duct extending to the expected location of the minor papilla. A smaller branch of the main pancreatic duct extended to the vicinity of the ampulla, but terminated inferior to the distal common bile duct without a confluence of the 2 structures.

**Figure 1.**
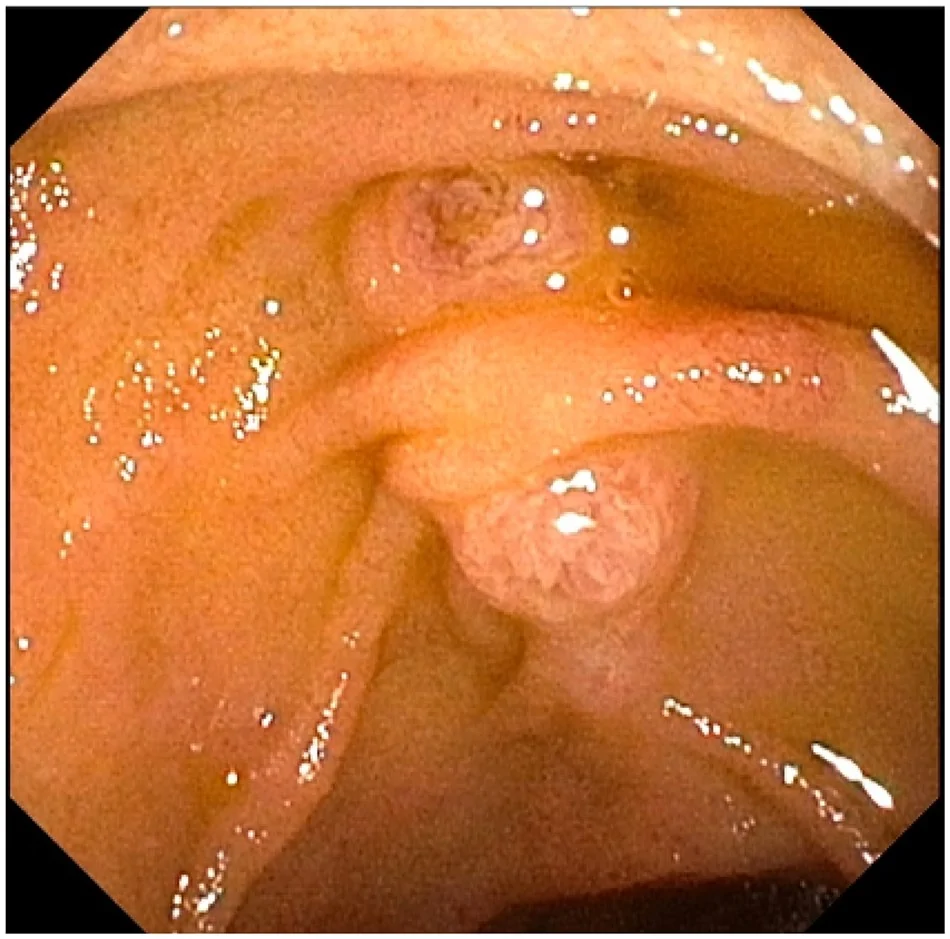
Endoscopic image demonstrating double ampulla anatomy.

## Discussion

To our knowledge, this is the first case of concurrent double ampulla and pancreas divisum anatomic variants described in the literature. Double ampulla anatomy occurs when non-union of the common bile duct and pancreatic duct leads to 2 distinct ampullae in the duodenum.^1^ This is a rare anatomic variant, occurring in approximately 0.18% of patients undergoing ERCP.^2^ A previous study found that common bile duct stones were more common in patients with double ampulla anatomy and hypothesized that associated sharp angulation and bile stasis may contribute to this.^3^ The presence of double ampulla anatomy may also lead to greater manipulation and cannulation of the duct of Wirsung, raising the risk of post-ERCP pancreatitis.^4^ While rare, proper identification and awareness of double ampulla anatomy are important to limit procedural risks of ERCP.

## Supplementary Material

gwaf025_Supplementary_Data

## Data Availability

There are no data associated with this manuscript.
